# Apple-core lesion of the colon: a case report

**DOI:** 10.4076/1757-1626-2-7275

**Published:** 2009-09-14

**Authors:** Ahmed Alzaraa, Kurzatkowski Krzysztof, Raphael Uwechue, Ming Tee, Chelliah Selvasekar

**Affiliations:** Department of General Surgery, Leighton HospitalCrewe, CheshireUK

## Abstract

**Introduction:**

The appearance of the apple-core lesion of the colon can be caused by several diseases.

**Case presentation:**

A male patient was referred to the surgical clinic with melaena and weight loss. Clinical examination and investigations confirmed an apple-core lesion of the colon. He underwent surgery, but unfortunately, died of a chest infection two weeks after the operation.

**Conclusion:**

This case shows the use of computed tomography in demonstrating the primary cause of iron deficiency anaemia, and at the same time, staging the disease for loco-regional metastasis.

## Case presentation

An 86-year-old white English man was referred to the surgical clinic in September 2007 for melaena and weight loss. He was anaemic on physical examination with no other clinical findings. He had a history of a left pneumonectomy in 1985 for a squamous cell carcinoma of the lung, Chronic Obstructive Airway Disease, Benign Prostatic Hypertrophy and diverticulosis. His Haemoglobin was 6.0 gm/L.

**Figure 1.**
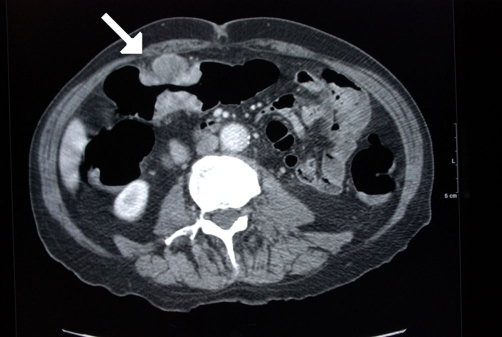
Abdominal Ct showing an apple-core lesion of the transverse colon (arrow) in an 86 years-old male patient.

**Figure 2.**
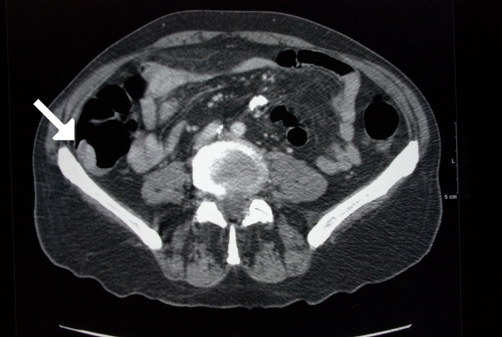
Abdominal CT showing a polyp in the ascending colon (arrow) in an 86 years-old male patient.

**Figure 3.**
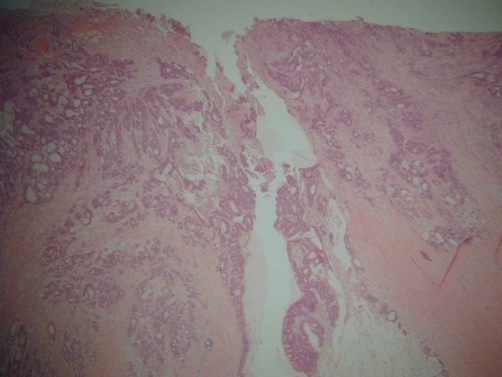
Low power view of the moderately differentiated adenocarcinoma of the colon in an 86 years-old male patient (x20).

**Figure 4.**
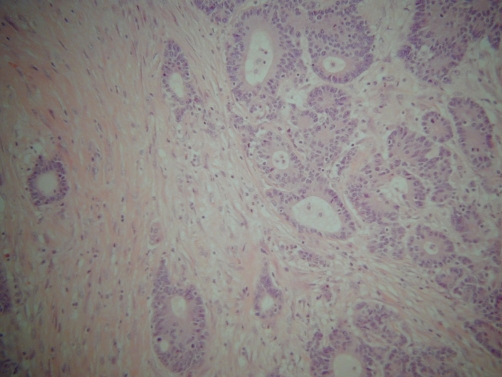
High power view of the moderately differentiated adenocarcinoma of the colon in an 86 years-old male patient (x40).

Abdominal CT (Figure 1) showed an apple-core stenosing tumour in the proximal transverse colon and a 2 cm intra-luminal lesion in the mid-ascending colon. He underwent a radical right hemicolectomy in November 2007. Macroscopic examination of the specimen revealed a 50 mm × 45 mm circumferential tumour in the large bowel, and invading the full thickness of the wall. The tumour was present at the serosal surface, and was situated 40 mm from the distal resection margin and 160 mm from the proximal resection margin. Other findings included full thickness (probably incisional) defect, 30 mm in maximum diameter that lied 80 mm proximal to the tumour and adjacent to a 24 mm sessile polyp (Figure 2). The background mucosa showed six polyps in total, measuring between 4 mm and 25 mm, and the largest lied 60 mm from the proximal resection margin. The terminal ileum was sliced through the wall at approximately 40 mm from the proximal end and was attached to the rest of the ileum by a strand of ileal tissue only. Microscopy showed moderately differentiated adenocarcinoma (Figures 3,4). The tumour had an infiltrating growth pattern with minimal lymphocytic infiltration at the advancing edge. The tumour was present at the serosa (pT4). An area suspicious of extramural lymphovascular invasion was also seen. The largest polyp was a severely dysplastic tubulovillous adenoma. The rest of the polyps were moderately dysplastic tubulovillous and tubular adenomas. One other hyperplastic polyp was also identified. Thirteen lymph nodes were identified and none of them showed metastatic deposits. All those findings confirmed an adenocarcinoma, Dukes’ B, pT4N0Mx. Unfortunately, his condition deteriorated after developing a chest infection and died two weeks after the operation.

## Discussion

The appearance of the apple-core lesion of the colon can be caused by several diseases. The differential diagnosis includes colonic adenocarcinoma, lymphoma, Crohn’s disease, chronic ulcerative colitis, ischaemic colitis, Chlamydia infection, tuberculosis, Helminthoma, Amoebiasis, Cytomegalovirus, villous adenoma, and radiosurgery such as high doses of Cyberknife used for treating unresectable abdominal malignancies, for example, pancreatic cancer [1-3].

**Figure 5.**
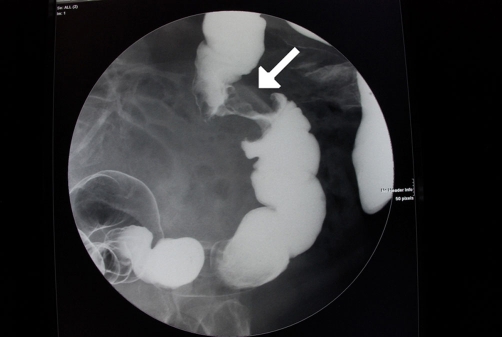
Apple-core lesion of the colon shown on barium enema (arrow).

**Figure 6.**
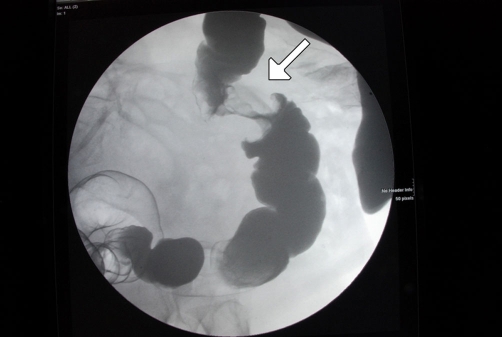
Apple-core lesion of the colon shown on barium enema (arrow).

**Figure 7.**
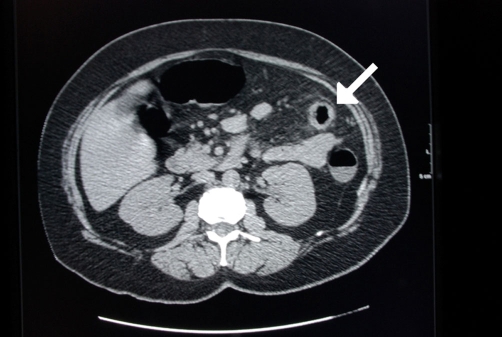
Abdominal CT showing a stricture of the sigmoid colon in ulcerative colitis (arrow).

Colonic carcinoma is usually detected on colonoscopy or barium enema for evaluating vague abdominal symptoms (Figures 5,6). The colonic stricture is usually 3 cm-4 cm in length and rarely exceeds 6 cm [4]. Mucosal irregularities and an eccentric lumen with overhanging shoulders characteristics of an apple-core lesion are suggestive of carcinoma, but histology remains the keystone in confirming the diagnosis [4,5]. Crohn’s stricture has a smooth lumen and tapered ends that fuse into the normal bowel, and is present in up to 25% of the cases of Crohn’s colitis. The strictures of ulcerative colitis are more frequent in the sigmoid colon (Figure 7) and are radiographically similar to those of Crohn’s disease [4].

**Figure 8.**
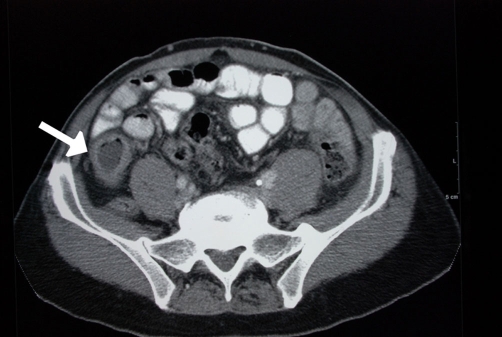
Abdominal CT demonstrating a stricture of intestinal tuberculosis at ileal region (arrow).

Intestinal tuberculosis occurs in the ileocaecal region in 90% of the cases. The stricture involves a longer segment of bowel, and is associated with nodular or ulcerated mucosa (Figure 8). In most cases, it has no one characteristic appearance [4,5]. Amoebiasis causes stricture in 2%-8% of its chronic cases. Its stricture is concentric with tapering ends, and is longer than that of a carcinoma, but when the shorter than that it is eccentric and may mimic a carcinoma [4]. Chlamydia trachomatis causes strictures and fistulas in the rectosigmoid, and may resemble carcinomas or Crohn’s disease [4].

**Figure 9.**
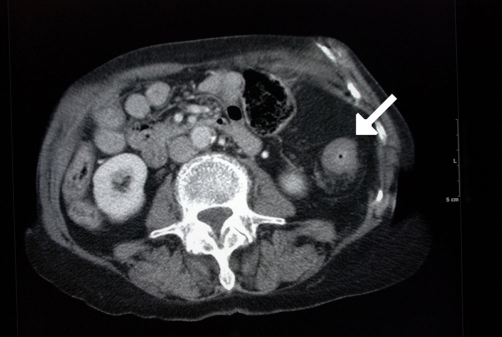
Abdominal CT showing an ischaemic stricture of the colon (arrow).

The ischaemic stricture has gradually tapering margins with concentric narrowing of the bowel lumen and intact mucosa (Figure 9). It usually involves distal transverse colon, the splenic flexure and the caecum [6]. Villous adenomas are mostly located below the peritoneal reflection (79% in the rectosigmoid or distal to it). Rarely, they may be found as high as the hepatic flexure [7]. On barium enema, they appear soft and produce sessile filling defects with irregular mucosal patterns known as reticular, granular, lacy, or feathery. This appearance is due to the collection of barium in the interstices of the adenomas ‘frond-like excrescences’ [8].

## Conclusion

In this case, we have demonstrated the value of CT scan in detecting a typical apple-core stricture as the cause of patient’s iron deficiency anaemia. This test provides a confirmatory radiological diagnosis and helps stage the disease with a single investigation. This may assist in reducing the delay between symptoms and treatment.

## Abbreviation

CT: computed tomography
